# Ion channel gene expressions in infertile men: A case-control study

**Published:** 2017-12

**Authors:** Serkan Carkci, Ebru Onalan Etem, Seda Ozaydin, Ahmet Karakeci, Ahmet Tektemur, Tunc Ozan, Irfan Orhan

**Affiliations:** 1 *Department of Urology, Faculty of Medicine, Firat University, Elazig, Turkey.*; 2 *Department of Medical Biology, Faculty of Medicine, Firat University, Elazig, Turkey.*

**Keywords:** Infertility, Male, Genes, Ion channels, Sperm

## Abstract

**Background:**

Infertility is described as not receiving pregnancy despite unprotected and regular sexual intercourse in a 1 yr period. It is detected by 15% of the couples. Male and female factor in the etiology may be detected in similar rates.

**Objective:**

The present study aims to investigate ion channel gene expression in semen samples of infertile male compared with fertile men.

**Materials and Methods:**

A total of 150 men who applied to the urology clinic due to infertility were divided into five equal groups: asthenozoospermia, oligozoospermia, oligoasthenoteratozoospermia, teratozoospermia, and normozoospermia (control). All paticipants were evaluated with Cation Channel Spermia (CatSper) 1, 2, 3, 4, Proton Voltage Gated Ion Channel1 *(Hv1),* Potassium Channel Subfamily U1 *(KCNU1*), and transmembrane protein (*TMEM16A)* gene expression in semen samples.

**Results:**

*“CatSper1, 4, HV1, KCNU1, *and* TMEM16A *gene expression were detected higher in the oligozoospermia group compared to the controls*. CatSper1, 2, 3, 4, KCNU1, *and *TMEM16A *gene expression in the asthenozoospermia group and* CatSper1, 2, 3, 4, KCNU1, *and* TMEM16A *gene expression in the teratozoospermia group were detected lower compared to the controls.* CatSper1, 4, HV1, *and* TMEM16A *gen expression were higher in the oligoasthenoteratozoospermia men than the controls while* CatSper3 *gen expression was detected as lower.”

**Conclusion:**

It was detected that these ion channels have an effect on sperm progressive motility and morphology. It may be considered that mutations in these ion channels may result in infertility.

## Introduction

The etiology of infertility remains still unclear by more than 50% of the couples wishing to achieve pregnancy (1). Elements, such as zinc, magnesium, copper, chlorine, potassium and calcium, are important for the maintenance of normal spermatogenesis, sperm maturation, DNA metabolism and repair and gene expression in germ cells (2). Spermatozoa mainly perform the transport of Na^+^, K^+^, Cl^-^ and Ca^+2^ ions through voltage gated and ligand gated ion channels. Recent investigations in knockout mouse models have identified many ion channel genes involved in spermatogenesis, and their mechanisms of action are currently being clarified. However, some of these findings have been directly applicable to humans because phenotype in mice does not always have direct counterparts in humans (1, 3). After ejaculation of the sperm cells into the female reproductive tract, the sperm cells enter a different micromilieu where ion concentrations, pH and osmotic pressures are changed (4). By the adaptation period, the sperm cells get involved in processes like capacitation, acrosome reaction and fertilization, where normal function of Ca+2, K+, Cl-, Gamma-Amino Butyric Acid and ion channels is required (5).

The major ion channels which may be found in the sperm cell are Cation Channel Spermia *(CatSper)*, Potassium Channel Subfamily U1 *(KCNU1*), Proton Voltage-Gated Ion Channel1 *(Hv1),* transmembrane protein *(TMEM16).* *CatSper 1, 2, 3, 4* are weakly voltage dependent and pH sensitive ion channels. They are permeable for Ca^2+^ ions and demonstrate sperm specific features. It was demonstrated that the localization of the CatSper ion channels are at the main part of the sperm flagellum. These ion channels are necessary for progressive sperm motility, sperm decomposition in the female reproductive tract, oocyte penetration and fertilization (3). Human spermatozoa are immotile in the male reproductive system and get activated by entering the female reproductive tract. Alcalization of the sperm cytoplasm is necessary for this activation process. The *Hv1* which is also located at the main part of the sperm's flagellum like *CatSper* plays a role by the induction of the intracellular alcalization and the activation of the spermatozoa. 

The *Hv1* also helps activation of *CatSper*ion channels. *SLO3 /KCNU1* is potassium voltage-gated ion channel and is also located in the main part of the sperm's flagellum. It was reported that *SLO3* is sensitive to both pH and the voltage level, plays a role by sperm capacitation and/or acrosome reaction and a mutation in the gene coding this ion channel may result in defective sperm activation and fertility. *SLO3* is shown to facilitate the potassium ions to move out of the sperm cell and to decrease the membrane voltage into more negative values. *SLO3 *has a vital function in the acquisition of normal morphology and sperm motility (1). *TMEM16* is a calcium activated chloride channel and is located in the head part of the spermatozoa, it is thought to have a role by induced acrosome reaction (6). 

In this study *CatSper1, 2, 3, 4, KCNU1, Hv1, *and *TMEM16* ion channel expressions were studied in infertile men with oligoasthenospermia, isolated asthenozoospermia, teratozoospermia, and oligozoospermia in comparison with fertile individuals with normal sperm parameters.

## Materials and methods

A total of 120 infertile men (19-55 yr) referred to the urology outpatient clinic between June 2014 and June 2015 were enrolled the study. And also 30 participants enrolled the study as control group Participants were divided into 5 groups (n=30/each) according to their semen analysis (2010 WHO criteria) (7): normozoospermia (control) oligozoospermia, asthenozoospermia, teratozoospermia (According to Kruger’s strict criteria normal morphology <4%), and oligoasthenoteratozoospermia groups. 

All men with varicocele, reproductive pathology in family history, and abnormal genetic profile were excluded from the study. The detailed anamnesis of the patients were recorded and physical examination and laboratory findings were evaluated.The age, duration and type of the infertility, method of contraception and frequency of weekly sexual intercourse, history of smoking, mumps, orchitis, prostatitis, epididymitis, and using assisted reproductive techniques was recorded for all participants. 

General physical examination and scrotal examination including epididymis, vas deferens, and plexus pampiniformis examination were performed by the same physican. Laboratory tests included blood follicle stimulating hormone (FSH), luteinizing hormone (LH), prolactin (PRL) levels, total testosterone (TT), and semen analysisFSH, LH, PRL,TT hormone parameters are measured with Advia Centaur XP ((Simens Healthcare Diagnostics, Germany) device with Siemens commercial kits by using chemo-luminescence method.

Patients with abnormal sperm parameters underwent a second semen analysis after a period of one month. Semen analysis was performed after 3 days of abstinence by the same andrology laboratory technician. Samples were scanned under 100X magnification with phase-contrast microscopy (Olympus CX41, USA) Number, motility and morphology of the sperms were studied under 200X magnification with the same microscope. 

**Methods used for ion channel assessment**

**Total** **RNA isolation**

RNA isolation is completed according to Georgiadis *et al* (8). Semen samples were centrifuged by 5000 g at a temperature of 4^o^C. The supernatant part was removed and the pellet was suspended in 1ml 0.5% Triton-X solution. The samples were kept in ice for 10 min for the lysis of the somatic cells. Then, the hypotonic Triton X solution was removed by centrifugation for 5 min at 5000 g and 4^o^C and the pellet was washed with PBS. Then, they were resolved in 1 ml Trizol. The solution was aspirated and ejected through a 30 G needle for 3 times, vortexed for 30 sec and then incubated at room temperature (24^o^C) for 5 min. The homogenised samples were centrifuged at 4^o^C by 12.000 g for 10 min and the supernatant part was removed. Lastly, 200 µl chloroform was added. The samples were vortexed for 30 sec and incubated at room temperature for 5 min. The liquid phase was transferred to a new tube and equal amount of isopropanol, one-tenth amount of 3M ammonium acetate and 1 µl glycogen was added. The samples were incubated at room temperature for 30 min and then centrifuged by 20.000 g at 4^o^C for 30 min. The supernatant part was removed and the pellet was washed with 75% ethanol. The samples were centrifugated by 12.000 g at 4^o^C for 5 min, ethanol was removed and the pellet was dried at room temperature then resolved in 20 µl water and a temperature of 58^o^C was applied for 15 min. RNA Assay Kit For Use With The Qubit® 2.0 Fluorometer (Invitrogen/ Molecular Probes) was used. The RNA amount was measured in μg/ml.

**Complementary DNA S** **ynthesis**

High- Capacity cDNA Reverse Transcription Kit (P/N: 4387406, Applied Biosystem, USA) was applied for cDNA synthesis. Reverse transcriptase reactions including RNA specimens with purified total RNA, 1× RT buffer, 0.25 mM each of dNTPs, 1 U/µl MultiScribe reverse transcriptase and 0.25 U/µl RNase inhibitor. The specimens were put into thermal cycler and incubated at 25^o^C for 10 min, at 37^o^C for 120 min, at 85^o^C for 5 min and were preserved at 4ºC. The consisting cDNA specimens were kept at-20^o^C (8).

**cDNA Amplif** **ication with Real Time Polymerase Chain Reaction (RT-PCR)**

The cDNA fragments generated by reverse transcription were amplified with RT-PCR by the presence of sequence-specific primers. The RT-PCR was performed on repeated cycling of three steps. By the preparation of the RT-PCR plate, cDNA samples of 2 μl were put into the wells. For every sample on ice 5 μl TaqMan Master Mix, 2.5 μl nuclease-free water and 0.5 μl primer hybridisation probe and component amounts calculated according to the sample size were placed into the Eppendorf tubes and vortexed. Eight μl of the prepared mixture was put on the cDNA samples on the plate and the plate was covered with optic adhesive tape. The samples were centrifuged with miniplate spinner for one minute in order to reduce generated bubbles and to achieve precipitation.

The normalization of the RNA specimens based on the TaqMan® Gene Expression Assays (Applied Biosystems, Foster City, CA, USA) for glyceraldehyde-3-phosphate dehydrogenase (GAPDH) endogenous controls. Real-time PCR was applied by using a standard TaqMan® PCR kit (P/N: 4370074, Applied Biosystem, USA) protocol on an Applied Biosystems 7500 Fast Thermal Cycler. The 10 µl PCR contained 1 µl RT product, 1× TaqMan® Universal PCR Master Mix, 0.5 µM TaqMan® gene specific assay mix (Applied Biosystems, Foster City, CA, USA). Gene expression levels were assesed by using Applied Biosystems 7500 Real-Time PCR system. GAPDH was applied as the control gene. The reactions were kept in a 96-well plate at 95^o^C for 10 min, followed by 40 cycles of 95^o^C for 15 sec and 60^o^C for 1 min. All reactions were repeated 3 times (8). Table I shows features and ID number of investigated genes.. 

**Ethical consideration**

Firat university local ethical committee approved the study protocol (30.10.2013/06/14). All participants signed the informed consent form.

**Statistical analysis**

All statistical analyses were performed using the SPSS statistical software package for Windows (version 22.0; IBM SPSS Inc., Chicago, IL, USA). SPSS package programme licensed by our institution (License Nr: 193.255.124.131). Post Hoc and student t-test were used to compare the groups according to demographic data, hormone levels, sperm motility and morphology. Correlation analysis was determined by using Pearson correlation analysis. The p˂0.05 was considered as statistically significant.

**Table I:** Properties and applied biosystem ID codes of ion channels

**Gene symbols**	**Localization**	**Number of exons**	**Number of aminoacids**	**Permeable ion**	**Assays IDs**
*CatSper1*	11p13.1	12	780	Ca^++^	Hs00364950_m1
*CatSper2*	15q15.3	14	528	Ca^++^	Hs00542505_m1
*CatSper3*	5q31.1	8	398	Ca^++^	Hs00604374_m1
*CatSper4*	1p36.11	10	472	Ca^++^	Hs01374398_m1
*Hv1/HVCN*	12q24.11	7	273	H^+^	Hs01032838_m1
*KCNU1/ SLO3*	8p11.23	27	1149	K^+^	Hs00380426_m1
*TMEM16A/ ANO1*	2q13.1	9	743	K^+^	Hs00216121_m1
*GAPDH*	8p11.23	27	1149	K^+^	Hs02758991_g1

CatSper: Cation channel, sperm associated

Hv1: Hydrogen voltage 1

HVCN: Hydrogen voltage gated channel

KCNU1/SLO3: Potassium channel subfamily U1

TMEM16A: Transmembrane protein 16A

ANO1: Anoctamin 1, calcium activated chloride channel

GADPH: Glyceraldehyde-3-phosphate dehydrogenase

Ca: Calcium

H: Hydrogen

K: potassium

## Results

The mean age of the 120 patients and 30 controls in the study was detected as 32.7±6.7 yr. Table II was given laboratory findings of the study groups. 

By comparison of the control with all of the abnormal sperm parameter groups, FSH, LH, levels and kruger, motilite findings was detected significantly differences (p=0.005, p=0.021, p<0.001 and p<0.001 respectively). By the comparison of the control with the oligo group, statistically significant difference is observed regarding to FSH, LH and Kruger parameters (p=0.042, p=0.023 and p=0.025, respectively). The comparison of asthenozoospermia group with the oligozoospermia group revealed a significant differentcence FSH AND sperm count (p<0.001 and p=0.021, respectively). Significanty difference has shown in terms of sperm count and morphology and by comparison of the asthenozoospermia group with the oligoasthenoteratozoospermia group (p<0.001 and p<0.001, respectively). 

When comparing asthenozoospermia group with the teratozoospermia group it revealed significant difference kruger (p<0.001) a in terms of sperm count (p>0.05) and kruger was detected significant difference of the oligozoospermia group with the oligoasthenoteratozoospermia group (p=0.003 and p<0.001, respectively). By comparison of oligozoospermia with the teratozoospermia group it revealed a significant difference in FSH, sperm count and kruger (p<0.001, p=0.034 and p<0.001, respectively). A significant difference was found by comparison of oligoasthenoteratozoospermia group with the teratozoospermia group in terms of the sperm count and kruger (p<0.001 and p=0.003, respectively).

There was no correlation detected between age, sperm count, FSH, LH, TT, PRL, morphology and motility levels in the control and oligozoospermia groups. A positive correlation was found between age and FSH, PRL and LH in the asthenozoospermia group (p=0.06; r=0.609, p=0.012; r=0.564, respectively). By the oligoasthenoteratozoospermia group, a positive correlation between age and FSH, morphology and age, kruger and FSH was detected and a positive correlation (p=0.015; r=0.655, p=0.023; r=0.580, p=0.038; r=0.579, respectively) and LH ile testereron arasonda negative korelasyon gözlendi (p=0.047; r=-0.609) a negative correlation between morphology and TT, age and motility and positive sperm count and LH correlation was detected in teratozoospermia group (p=0.12; r=-0.626, pp=0.049; r=-0.376, p=0.05; r=0.514).

In our study, we have detected a decrease in the gene expression of CatSper1, CatSper2, CatSper3, CatSpe4, TMEM16A and KCnu1 gene expression by asthenozoospermia patients were detected as lower compared to the controls (p=0.022, p<0.001, p<0.001, p=0.007, p=0.016, p<0.001, respectively) and no significant difference in the other analyzed gene expression. CatSper1, CatSper3 gene expression by teratozoospermia patients were detected as lower compared to the controls (p<0.001, p<0.001). No significant difference in other gene expressions was detected in teratozoospermic group. By the oligozoospermia group an increase was observed in CatSper1, CatSper4, Hv1, SLO3 controls (p<0.001, p=0.003, p<0.001, p=0.016, respectively). whereas a decrease was found in TMEM16A gene expression. by oligoasthenoteratozoospermia patients were detected as higher (p<0.001 for all genes).

**Figure 1a:**
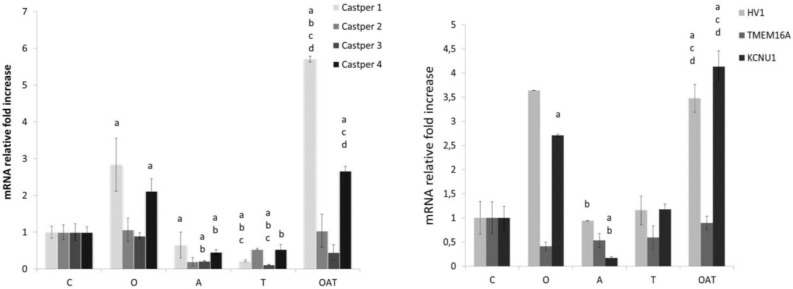
Quantitative real-time PCR validation of transcriptome data for selected ion channel genes. The mRNA fold increase graphic of Ca+2 permeable CatsPer1, 2, 3, and 4 genes. **b.** The mRNA fold increase graphic of *SLO3 (KCNU1), TMEM16A*, and *HVv1.* GAPDH was used as an internal control (see the Experimental section). Results are means±S.E.M. from three independent experiments. T-test was performed for statistical analysis (n=30 for each group); different superscript letters indicate significant differences (p<0.05), a: vs control group; b: vs oligozoospermic group; c: vs asthenozoospermic group; d: vs teratozoospermic group. (student’s test).

The oligoasthenoteratozoospermia group revealed an increase in CatSper1, Castper4, Hv1 and KCNu1 gen expressions higher (p<0.001 for all genes), no difference in gene expressions of CatSper2 and KCNU1 and a decrease in CatSper3 gene expression. Figure 1 shows mRNA relative fold increase graphic of CatSper 1, CatSper 2, CatSper 3, CatSper 4, Hv1, TMEM16A and SLO3 genes.

Between sperm count and sperm progressive motility with CatSper1 gene expression were found negative correlation (p=0.00 p<0.001, p=0.041). We found positive correlation between CatSper3 gene expression with FSH level, sperm count, progressive motility and Kruger (p=0.040, p=0.002, p=0.001, p<0.001). Between sperm count, progressive motility and TMEM16a expression was detected negative correlation (p=0.026, p=0.00 p<0.001, respectively). Between KCNU1 gene expression and sperm count was detected negative correlation (p=0.023). The other correlations between gene expressions with sperm count, progressive motility and morphology are shown in table III.

**Table II:** Laboratory findings of the study groups

	**Control**	**Oligozoospermia**	**Asthenozoospermia**	**Teratozoospermia**	**Oligoastheno-** **teratozoospermia**	**p-value**
Age (yr)	31.6 ± 6.1	34.2 ± 7.0	33.2 ± 8.1	31.5 ± 4.9	34.8 ± 7.7	P= 0.369 F= 1.082
FSH (mIU/ml)	3.4 ± 1.4	8.7 ± 5.5^a^	5.0 ± 2.9 ^a^^ ,^^b^	5.2 ± 2.5 ^a^	6.8 ± 3.1 ^a^^, ^^c^	P= 0.001 F= 5.342
LH (mIU/ml)	3.3 ± 1.1	5.6 ± 3.9 ^a^	4.5 ± 1.7 ^a^	4.6 ± 2.0 ^a^	5.1 ± 2.9 ^a^	P= 0.128 F= 1.854
PRL (ng/ml)	8.3 ± 4.2	8.4 ± 2.4	7.8 ± 2.9	9.6 ± 4.9	8.1 ± 4.7	P= 0.751 F= 0.478
T.T. (ng/dl)	428.6 ± 125.7	410.5 ± 236.4	402.7 ± 130.1	426.0 ± 141.4	369.6 ± 107.3	P= 0.875 F= 0.303
Sperm Count (10^6^)	63.0 ± 27.02	5.5 ± 3.3	54.5 ± 31.1 ^a^^, ^^b^	41.0 ± 21.9 ^a^^, ^^b^^,^^c^	9.5 ± 4.0 ^a^^, ^^c^^,^^d^	P <0.001 F= 28.764
Motility (%)	43.2 ± 7.1	43.9 ± 9.9 ^a^	21.2 ± 6.8 ^b^	41.0 ± 7.2 ^a^^, ^^b^^, ^^c^	15.6 ± 8.5 ^b^^, ^^d^	P<0.001 F= 54.91
Kruger (%)	4.4 ± 0.5	3.9 ± 0.6 ^a^	4.0 ± 0.7 ^a^	1.5 ± 0.5 ^a^^, ^^b^^, ^^c^	0.8 ± 0.8 ^a^^, ^^b^^, ^^c^^, ^^d^	P<0.001 F= 133.939

One Way ANOVA was used to compare the groups according to demographic data, hormone levels, sperm motility and morphology.

FSH: Follicle stimulating hormone

LH: Luteinizing hormone

PRL: Prolactin

TT: Total testosterone

a : vs control group;

b : vs oligozoospermic group;

c : vs asthenozoospermic group;

d : vs teratozoospermic group.

**Table III:** Correlations between gene expressions and hormones and sperm properties

	**FSH**	**LH**	**Prolactin**	**Testosterone**	**Sperm count (×10** ^6^ ** /ml)**	**Progressive motility (%)**	**Kruger**
*Casper 1*	0.184 p= 0.112	0.092 p= 0.437	-0.065 p= 0.585	-0.099 p= 0.400	-0.366 p<0.001	-0.188 p= 0.041	0.046 p= 0.0625
*Casper 2*	-0.086 p= 0.462	0.027 p= 0.819	0.169 p= 0.150	0.077 p= 516	0.05 P= 0.57	-0.015 p= 0.871	0.061 p= 0.511
*Casper 3*	-0.236 p= 0.040	-0.06 p= 0.959	0.041 p= 0.730	0.178 p= 0.129	0.272 p= 0.002	0.299 p= 0.001	0.357 p<0.001
*Casper 4*	0.201 p= 0.081	0.081 p= 0.494	-0.075 p= 0.525	0.000 p= 0.998	-0.300 p= 0.001	-0.017 p= 0.857	0.076 p= 0.0412
*Hv1*	0.101 p= 0.384	-0.46 p= 0.698	0.023 p= 0.845	0.075 p= 0.523	-0.071 p= 0.432	0,.092 p= 0.318	0.112 p= 0.229
*TMEM16A*	0.06 p= 0.609	0.030 p= 0.801	-0.054 p= 0.650	-0.104 p= 0.377	-0.198 p= 0.026	-0.365 p<0.001	0.077 p= 407
*KCNU1*	0.056 p= 0.631	0.151 p= 0.198	0.143 p= 0.226	0.015 p= 0.896	-0.202 p= 0.023	0.139 p= 0.131	-0.118 p= 0.204

Correlation analysis was determined by using Pearson correlation analysis. The value p˂0.05 was considered as statistically significant.

CatSper: Cation channel, sperm associated

Hv1: Hydrogen voltage 1

TMEM16A: Transmembrane protein 16A

KCNU1: Potassium channel, subfamily U, member 1

FSH: follicle stimulating hormone

LH: luteinizing hormone

C: Control group

O: Oligozoospermic group

A: Asthenozoospermic group

T: Teratozoospermic group

OAT: Oligoasthenoteratozoospermic group.

## Discussion

We have detected statistically significant differences in the ion channel gene expressions by impaired progressive motility and morphology patients compared to the control.The incidence of male factor is reported as 30% by the etiology of infertility (9). Even by normal sperm parameters the etiology of infertility remains still unclear by more than 50% of the couples wishing to achieve pregnancy and this phenomenon is called idiopathic infertility (1). Ion channels are one of the new research topics and are considered to have an important role in the etiology of idiopathic infertility (10). It has been shown that loss of function in the ion channels may impair sperm physiology by causing abnormal capacitation, loss of hyperactivation and impaired acrosome reaction (1). Even by the presence of complete normal sperm parameters, fertilization may fail. For this reason, it is of big importance to understand the ion channel functions on the sperm cell because it may lead to develop new treatment strategies for idiopathic infertility. 

*CatSper* is a sperm-specific, weak voltage-dependent calcium selective channel and is pH sensitive. It acts by the control of the positive loaded calcium ions into the sperm cell and is necessary for sperm hyperactivation and fertilization. It was shown that *CatSper* ion channels are located on the main part of flagellum by rat and human. *CatSper* is a heterotetrameric calcium channel and is consisted of 4 different pore forming alfa subunits; *CatSper 1, 2, 3, 4,* and *3* auxiliary subunits called *CatSper β (beta),* *CatSper γ (gamma)* and *CatSper δ (delta)* (3). Ren and his colleagues have shown the necessity of CatSper presence for normal sperm motility and oocyte penetration. In studies consisting of *CatSper*-/-transgenic and normal rats it was detected that *CatSper-*/-sperm revealed weak vitality, decreased motility and curling in the tail part. Studies performing computer-assisted sperm analysis have shown impairment in the forward progressive motility and decrease in swimming speed by *CatSper*-/-sperm (11). 

In another study, fertilisation capacity of *CatSper*-/- was evaluated in-vitro and it was shown that *CatSper*-/-sperm were not able to fertilize the oocyte while normal sperm cells revealed a fertilisation rate of 81%. It is reported that some *CatSper*-/-sperm were able to adhere but not penetrate to the oocyte and the study has also shown that fertilisation occurs in oocytes with enzymatically removed zona pellucida by both *CatSper*+/+ and *CatSper*-/-rat sperm which may confirm strongly that *CatSper* is necessary for penetration (1). In a study of Tamburrino *et al*, it was reported that *CatSper1* gene expression was decreased in spermatozoa from asthenospermic patients. The study also showed a decrease in progressive motility and acrosome reaction (12).

Another study revealed that *CatSper1*-/-and *CatSper2*-/-spermia are not able for hyperactivation during capacitation process (3). Avenarius and colleagues have found a correlation between *CatSper1 *and *CatSper2* mutation and asthenoteratospermia (14). In a study of Bhilawadikar and colleagues, it was shown that *CatSper2* expression was decreased by asthenozoospermic patients (15). Hildebrand and colleagues have detected that by the absence of *CatSper2 *expression sperm malformation was present by 88% while short sperm head and curling flagellum was present by 30% which they were correlated with the decrease in sperm motility (16). 

Jin and colleagues have stated that *CatSper3*-/- and *CatSper4*-/-male rats were infertile even they were showing normal mating attitude. In their study, they have shown that *CatSper3*-/- and *CatSper4*-/-rats were infertile despite their normal sperm count and motility. The same study has reported that hyperactivation during capacitation was observed 30 min after incubation at room temperature by normal spermatozoa but first motility was detected at 2 hr after incubation by *CatSper3*-/- ve *CatSper4*-/- sperm cell. This result supported that functional *CatSper3 *and *CatSper4* may be necessary for hyperactivation of sperm during capacitation (17). By scanning of current literature only one study about *CatSper* ion channels in human was found. This study reported a correlation between *CatSper1* mutation and idiopathic asthenospermia (10). 

In our study we have reported a decrease in *CatSper1* and *CatSper2* gene expression by asthenozoospermia and teratozoospermia groups. The gene expression of *CatSper1 *revealed an increase by oligozoospermia and oligoasthenoteratozoospermia groups where as *CatSper2* gene expression revealed no significant change by oligozoospermia and oligoasthenoteratozoospermia groups. The study also revealed that presence of *CatSper1 *and *CatSper2* gene expression may be positively correlated with hyperactive motility and morphology. *CatSper3* gene expression has shown a decrease in the asthenozoospermia, teratozoospermia and oligoasthenoteratozoospermia groups compared to the control but no significant change in the oligozoospermia group. *CatSper3* ion channel was also positively associated with hyperactive motility and morphology. In our study we detected a decrease of *CatSper4 *gene expression in asthenozoospermia and teratozoospermia group and an increase in the oligozoospermia and oligoasthenoteratozoospermia groups. Our study also revealed a relation between *CatSper4* and hyperactive motility and morphology in a positive way. In the light of all these data revealed in our study we consider that *CatSper* ion channels may play an important role in the sperm physiology. 

In this study, we have detected a significant increase of *Hv1* gene expression in the oligozoospermia and oligoasthenozoospermia groups, an insignificant decrease in the asthenozoospermia group and no significant change in the teratozoospermia group compared to the control. As we have mentioned in the introduction part, human spermatozoa are activated by alcalization of the sperm cytoplasm and the *Hv1* gene is responsible for the induction of the intracellular alcalization by extracellular proton transfer and the activation of the spermatozoa. *Hv1* also activates the *CatSper* ion channels and plays a role by sperm hyperactivity. Since *Hv1* gene may not be found by rats, there is no sufficient data about detailed function of this gene but according to the known mechanisms regarding the function, we can predict that a potential *Hv1* ion channel mutation may result in infertility in human (18). 

*SLO3/KCNU1* is a potassium voltage-gated ion channel and is specific to the testicular tissue regarding some studies made by rats. *SLO3/KCNU1* is localized on the main part of the sperm’s flagellum (1*). KSper/SLO3* is considered as responsible for the potassium transfer and pH depended potassium conductivity in rat sperm cell. A physiologic ionic gradients of sperm with normal type KSP is *SLO3* is hyperpolarized, while *SLO3/KCNU1*-/-sperm is depolarized (19). Santi and colleagues have stated that *SLO3* can only be found by mammals and is specific for testis. In this study, they used *SLO3*/ *KCNU1* knock-out rats and showed a deficit in pH sensitive potassium flow by testicular sperm cell where no fertilization was observed after mating of these mutant homozygote male rats with healthy female rats. Studies revealed that *SLO3/KCNQ1* ion channels are the main responsible ion channels for hyperpolarization during capacitation and no other ion channel may substitute them. It has also shown that mutant sperm cells are depolarized after capacitation. *SLO3/KCNU1* mutant sperm cells may reach an acrosome reaction after termination of hyperpolarization but could not achieve the zona pellucida penetration. The Osmotic milieu is also an important issue for the sperm flow towards the oocyte. Potassium and *SLO3/KCNU1* ion channels play a role in this osmotic volume regulation. By osmotic volume dysregulation, the sperm cell swells and morphological changes occur. These changes may prevent the sperm cell to adhere to the oocyte and result in infertility (20). 

In our study, we detected an increase of *SLO3/KCNU1* gene expression in the oligozoospermia and oligoasthenoteratozoospermia groups and a decrease in the asthenozoospermia group compared with the control. No significant change was observed in the teratozoospermia group regarding the *SLO3/KCNU1* gene expression. *SLO3/KCNU1* gene expression decreased in the oligozoospermia, asthenozoospermia and teratozoospermia groups compared to the control but no significant change was observed in the oligoasthenoteratozoospermia group. Regarding these data, we can say that *SLO3/KCNQ1* gene expression may be specifically effective by progressive motility of the sperm cell. *TMEM16A* is a calcium-activated chloride channel located in the head part of the human sperm cell. The role of *TMEM16A* in the sperm capacitation and zona pellucida induced acrosome reaction was shown in former studies (21). Zanetti and Mayorga stated that *TMEM16A* is responsible for regulation of cell volume and plays an important role by reducing the distance between the outer acrosomal membrane and plasma membrane and facilitating the acrosome exocytosis (22). In our study, we have observed an increase of *TMEM16A* gene expression in oligozoospermia and oligoasthenoteratozoospermia groups and a decrease in the asthenozoospermia and teratozoospermia groups compared to the control.

## Conclusion

According to the results we have achieved in this study, we assume that a decrease in the function of the ion channels may be related to male infertility especially by asthenozoospermic and teratozoospermic patients. There is a need for detailed evaluation in future studies with larger numbers to investigate this mentioned relation by oligozoospermia and oligoasthenoteratozoospermia patients. We believe that complete clarification of the ion channel functions and developing activator/inhibitor molecules targeting these channels could bring therapeutic options for patients with idiopathic infertility into existence. Also, these activator/inhibitor molecules could affect solely on sperm cells and may be the precursors of drugs providing male contraception.
